# Neural evidence for non-orofacial triggers in mild misophonia

**DOI:** 10.3389/fnins.2022.880759

**Published:** 2022-08-09

**Authors:** Heather A. Hansen, Patricia Stefancin, Andrew B. Leber, Zeynep M. Saygin

**Affiliations:** Department of Psychology, The Ohio State University, Columbus, OH, United States

**Keywords:** misophonia, resting-state connectivity, fMRI, sensorimotor cortex, orofacial, finger-tapping

## Abstract

Misophonia, an extreme aversion to certain environmental sounds, is a highly prevalent yet understudied condition plaguing roughly 20% of the general population. Although neuroimaging research on misophonia is scant, recent work showing higher resting-state functional connectivity (rs-fMRI) between auditory cortex and orofacial motor cortex in misophonia vs. controls has led researchers to speculate that misophonia is caused by orofacial mirror neurons. Since orofacial motor cortex was defined using rs-fMRI, we attempted to theoretically replicate these findings using orofacial cortex defined by task-based fMRI instead. Further, given our recent work showing that a wide variety of sounds can be triggering (i.e., not just oral/nasal sounds), we investigated whether there is any neural evidence for misophonic aversion to non-orofacial stimuli. Sampling 19 adults with varying misophonia from the community, we collected resting state data and an fMRI task involving phoneme articulation and finger-tapping. We first defined “orofacial” cortex in each participant using rs-fMRI as done previously, producing what we call resting-state regions of interest (rsROIs). Additionally, we functionally defined regions (fROIs) representing “orofacial” or “finger” cortex using phoneme or finger-tapping activation from the fMRI task, respectively. To investigate the motor specificity of connectivity differences, we subdivided the rsROIs and fROIs into separate sensorimotor areas based on their overlap with two common atlases. We then calculated rs-fMRI between each rsROI/fROI and *a priori* non-sensorimotor ROIs. We found increased connectivity in mild misophonia between rsROIs and both auditory cortex and insula, theoretically replicating previous results, with differences extending across multiple sensorimotor regions. However, the orofacial task-based fROIs did not show this pattern, suggesting the “orofacial” cortex described previously was not capturing true orofacial cortex; in fact, using task-based fMRI evidence, we find no selectivity to orofacial action in these previously described “orofacial” regions. Instead, we observed higher connectivity between finger fROIs and insula in mild misophonia, demonstrating neural evidence for non-orofacial triggers. These results provide support for a neural representation of misophonia beyond merely an orofacial/motor origin, leading to important implications for the conceptualization and treatment of misophonia.

## Introduction

Imagine experiencing the same sense of anxiety, panic, or rage you feel toward the sound of nails scraping chalkboard to innocuous soft sounds in the environment, like chewing, breathing, or tapping. This is the reality for individuals with misophonia, a highly prevalent yet understudied disorder of sound processing. A consensus definition describes misophonia as a decreased sound tolerance to specific sounds or stimuli associated with the sounds, resulting in strong negative emotional, physiological, and behavioral responses not seen in other people (Swedo et al., 2021). Anecdotal reports from sufferers reveal serious daily impairments attributable to misophonia–job instability, deteriorating relationships, suicidal thoughts (Edelstein et al., 2013; Rouw and Erfanian, 2017; Swedo et al., 2021)–yet the condition is severely understudied with mechanisms vastly unknown.

At the time of this writing, 105 peer-reviewed misophonia articles exist on PubMed. Only seven of these articles, however, investigate this disorder using magnetic resonance imaging (MRI). MRI research enables a non-invasive and *in vivo* assessment of pathophysiology, neural mechanisms, and treatment strategies that have been instrumental to understanding other disorders (Dijkhuizen and Nicolay, 2003). In fact, neural markers based on functional magnetic resonance imaging (fMRI) during rest have been identified and proposed for various psychopathologies, such as obsessive-compulsive disorder (OCD) (e.g., Takagi et al., 2017), schizophrenia (e.g., Chahine et al., 2017; Wang et al., 2020), and bipolar disorder (e.g., Magioncalda et al., 2015; Wang et al., 2020). Are there neural markers for misophonia?

One of the first fMRI studies of misophonia found that individuals with misophonia showed significant differences as compared to healthy controls in the anterior insular cortex, specifically when presented with triggering auditory stimuli (Kumar et al., 2017). The anterior insular cortex has been implicated in a wide variety of functions, including subjective evaluation of pain (Brooks et al., 2002), goal-directed attentional control (Eckert et al., 2009; Nelson et al., 2010), interoception (Wang et al., 2019), and processing of disgust (Krolak-Salmon et al., 2003). Other fMRI work showed increased activation in the insula, anterior cingulate cortex, and superior temporal cortex (i.e., auditory cortex) in misophonia participants when presented with video clips depicting triggering actions, as compared to generally aversive actions or neutral actions (Schröder et al., 2019).

Previous work has also explored connectivity differences in misophonia. A recent study used diffusion weighted imaging (DWI) (a measure of structural connectivity and white matter tracts) and found that, as compared to healthy controls, individuals with misophonia had greater white matter volumes in the inferior fronto-occipital fasciculus, anterior thalamic radiation, and corpus callosum (Eijsker et al., 2021a). Additionally, several misophonia studies have explored functional connectivity, measured by correlating the fMRI activation of various regions of interest (ROIs) across time either during a task or while the brain is at rest, effectively measuring to what extent the ROIs spontaneously activate together. One study noted significant functional connectivity associated with misophonia between the anterior insular cortex and (a) posterior cingulate cortex/precuneus; (b) ventromedial prefrontal cortex; (c) hippocampus; and (d) amygdala (Kumar et al., 2017), a subcortical structure often implicated in emotion processing and regulation (Phillips et al., 2003; Ochsner et al., 2012). Another found increased functional connectivity in misophonia (a) between the amygdala and cerebellum and (b) within the lateral occipital/fusiform area of the ventral attention network (Eijsker et al., 2021b).

Most recently, Kumar et al. (2021) introduced a new hypothesis about the neural origins of misophonia using resting-state functional connectivity (rs-fMRI) within motor cortex, motivated by the use of mimicking movements as a common coping mechanism for sufferers (Edelstein et al., 2013). Mirror neurons in motor cortex would presumably be activated simply by seeing or hearing sensory input (e.g., the sound of chewing would evoke activity within the part of motor cortex responsible for chewing motions, even when performed by others) (see “audiovisual mirror neurons,” Kohler et al., 2002). (Kumar et al., 2021) therefore investigated the connectivity between auditory cortex (where sound is processed) and orofacial motor and premotor cortex (where chewing motions originate). Their data show that the orofacial region within the ventral premotor cortex is more strongly connected to the planum temporale and to the anterior insula in individuals with misophonia compared to healthy controls. The planum temporale functions as higher-level auditory cortex, often associated with speech comprehension (Shapleske et al., 1999) and the analysis of many types of complex sounds more broadly (Griffiths and Warren, 2002). Since the ventral premotor cortex is thought to be a key hub of the mirror neuron system (Rizzolatti and Craighero, 2004; Iacoboni and Dapretto, 2006; Kumar et al., 2021), conclude that misophonia is the result of hyperactivity of the mirror neurons in orofacial motor cortex, suggesting that the action of the trigger person is more important than the sound that is produced and providing what they call the “motor basis of misophonia.”

While these previous studies provide a foundation for future neuroimaging research on misophonia, there remain numerous gaps in this literature. First, the two task-based fMRI experiments on misophonia so far have assumed that misophonia is mainly an aversion to oral/nasal sounds. This is reflected both in the types of participants eligible for their studies and in the sound stimuli used in their tasks. For instance, Kumar et al. (2017, 2021) specifically recruited misophonic individuals who had oral/nasal sounds as triggers, then exclusively used human-produced oral/nasal sounds to comprise their “trigger” category. Schröder et al. (2019), Eijsker et al. (2021a,b) used in-house diagnostic criteria to assess their participants, which requires that human-produced oral/nasal sounds be a trigger to be diagnosed (Schröder et al., 2013; Jager et al., 2020). However, it is clear from both anecdotal self-reports and clinical interviews (e.g., Hadjipavlou et al., 2008; Edelstein et al., 2013; Ferreira et al., 2013; Johnson et al., 2013; Neal and Cavanna, 2013; Jastreboff and Jastreboff, 2014; Webber et al., 2014) as well as large-scale sound bank experiments employing machine learning (Hansen et al., 2021) and a consensus based on meta-analysis (Swedo et al., 2021) that individuals with misophonia are bothered by more than just oral/nasal sounds; restricting the condition to study just those triggers is likely to miss important findings.

Further, previous work may be biased by the construction and discussion of ROIs. For instance, Schröder et al. (2019) defined and analyzed activation in the entire insula but spoke of their significant results as “confirming” the Kumar et al. (2017) anterior insula finding. Kumar et al. (2021) used only the anterior insula as a seed region based on their prior results, sidestepping any role the posterior insula might play, despite the posterior insula’s known involvement in sensorimotor and auditory processing (see Uddin et al., 2017). Interestingly, Kumar et al. (2021) built their claims around an orofacial motor region, defined by “the part of vPMC [PMv] which showed stronger connectivity to planum temporale in resting-state.” Given the planum temporale is known for processing higher level auditory information, it is not clear why the part of motor cortex most strongly connected to auditory cortex would be selectively related to the mouth and face. Moreover, previous research may have encountered common issues with ROI-based connectivity analyses. For example, defining an ROI using functional connectivity and then analyzing functional connectivity of that same ROI to depict differences between misophonia and control groups, while orthogonal, is a circular analysis that may distort results when performed in the same sample (Kriegeskorte et al., 2009); when defined in one sample and overlaid onto a separate sample, circularity is avoided but individual variability in connectivity patterns is washed out (e.g., see Supplementary Figure 2 for example individual variability in ROI definition). Using task-based fMRI instead to localize an orofacial motor area and then exploring its connectivity with an rs-fMRI analysis on independent data within the same subject would remove these potential biases.

Lastly, the previous fMRI studies draw definitive conclusions from data that would benefit from stronger control conditions and tests of specificity. For example, Schröder et al. (2019) contrasted activation while watching trigger videos–which included both audio and visual stimulation–with a neutral condition in which videos depicted soundless activities (which would not elicit auditory activation like the trigger videos would). One cannot therefore conclude that “misophonia is associated with altered brain activity in the auditory cortex” because it may simply be a result of experimental design. Similarly, Kumar et al. (2021) focused on orofacial cortex within PMv, without exploring either (a) orofacial cortex in other motor/sensory regions (e.g., orofacial cortex defined in PMd), or (b) cortex representing non-orofacial body parts. This lack of dissociation begs for further research to make a more definitive claim that misophonia has a “motor basis.”

The present study seeks to fill in these important gaps and help clarify some of the seemingly conflicting claims about which particular brain regions and/or connections are responsible for misophonia. Our first objective was to theoretically replicate the results of Kumar et al. (2021), using an orofacial region that is functionally defined from task-based fMRI instead of estimated from resting-state connectivity. We know that functionally defining ROIs is more ecologically valid and better captures individual variability in cortical locations (Swallow et al., 2003; Saxe et al., 2006). If this functionally defined orofacial region likewise shows higher connectivity to planum temporale and insula in individuals with misophonia compared to controls, we can be more confident in the motor-basis finding. Second, we sought to investigate the selectivity of sensorimotor orofacial involvement in misophonia. Kumar et al. (2021) restricted their claims to orofacial motor and premotor regions, but given the high variation in experienced misophonia triggers, we expand the present analyses to include (a) a broader portion of sensorimotor cortex, and (b) cortex functionally linked to finger tapping.

## Materials and methods

### Participants

Twenty adults participated in this study. One adult was excluded for excessive motion (see Section “Resting state” below), resulting in nineteen adults (14 females, 5 males, mean age = 25.6) included in the present analyses. Of the nineteen adults, five identified as Asian, two as Middle Eastern, one as Latino, and the rest as Caucasian. All participants were recruited via advertisements on social media, flyers, and study websites. Participants were part of a larger ongoing longitudinal study of brain development and were paid a total of $30 for participating in the neuroimaging protocol.

All experimental methods were approved by The Ohio State University Institutional Review Board, and all participants gave informed consent to participate.

### Misophonia questionnaires

Each participant’s level of misophonia was determined using three misophonia assessment surveys available at the time of data collection. All participants completed the Misophonia Activation Scale (MAS-1) (Fitzmaurice, 2010), the Misophonia Assessment Questionnaire (MAQ-2) (Johnson, 2014), and the Amsterdam Misophonia Scale (A-MISO-S) (Schröder et al., 2013). Using a composite score that equally weighted the three misophonic assessment surveys (see Supplementary Table 1), with higher scores denoting more severe misophonia, the 19 participants had misophonia scores ranging from 0 to 37.3 out of 100.

For analyses comparing group means, we recreated the binary group division of Kumar et al. (2021) by splitting our sample *post hoc* using a score of 20 as cutoff, resulting in seven individuals with higher misophonia scores (mean = 28.7, range = 20.5–37.3; four females, three males; mean age = 24.2) and twelve individuals with lower misophonia scores (mean = 9.6, range = 0–17.7; nine females, three males; mean age = 26.4). This subdivision was further supported by individual scores on the A-MISO-S (Supplementary Table 1) and the suggested subdivisions provided by the authors (Schröder et al., 2013): individuals in the “higher misophonia” group all scored above 4 on the A-MISO-S (Mean = 7.7, SD = 1.1, Range = 6–9), corresponding with “mild” misophonia, whereas individuals in the “lower misophonia” group all scored below 4 (Mean = 2.2, SD = 1.5, Range = 0–4), corresponding with “subclinical” misophonia. For comparison, the misophonia group in Kumar et al. (2021) scored an average of 15.5 (SD = 3.4) on the A-MISO-S, corresponding with “severe” misophonia; scores are not reported for the control group.

Additionally, to probe any comorbid effects with other psychopathologies, all participants completed the Obsessive Compulsive Inventory-Revised (OCI-R) (Foa et al., 2002) and Depression Anxiety Stress Scale-21 (DASS-21) (Lovibond and Lovibond, 1995).

### Neuroimaging procedure

#### Acquisition

##### Scan parameters

All neuroimaging data were acquired at the Center for Cognitive and Behavioral Brain Imaging at The Ohio State University on a Siemens 3T Prisma scanner using a 32-channel head coil. A 3D magnetization-prepared rapid acquisition with gradient echo (MPRAGE) structural scan was acquired on all participants with high-resolution (1 mm^3^). Resting-state MRI data were acquired with a scan lasting approximately 10 min (TE = 28 ms, TR = 1000 ms, voxel size = 2 mm × 2 mm × 3 mm, flip angle = 61°, 56 slices, 580 volumes). Task fMRI data were also acquired on all participants (TE = 28 ms, TR = 1000 ms, voxel size = 2 mm × 2 mm × 3 mm, 56 slices, 186 volumes).

##### Functional magnetic resonance imaging task

As part of a larger neuroimaging protocol studying speech and language development, all participants completed an articulatory localizer consisting of alternating blocks of phoneme speech production, finger-tapping, and rest. At the beginning of each block, an image of a mouth or a hand was shown on the monitor. When shown the image of the mouth, participants were instructed to physically vocalize the syllables “BA GA RA DA” continuously until the image of the mouth was removed from the screen. When shown the image of the hand, participants were instructed to tap their fingers one at a time, from forefinger to pinky and back again, continuously until the hand was removed from the screen. The instruction image was presented at the start of each block for 2 s. Each of the two conditions was presented for 16 s blocks, and each condition block was presented four times per run. All participants included in the present study completed at least one run of this articulatory localizer; 11 participants completed two runs.

#### Pre-processing

##### Resting state

Resting-state pre-processing was performed using Freesurfer’s FS-Fast pre-processing pipeline.^1^ Framewise displacement was calculated for use as a motion regressor. Masks of white matter, cerebrospinal fluid (CSF), and subcortical structures were then generated in each participant’s native space. Next, spatial smoothing was performed using the subcortical mask, and functional data were interpolated over motion spikes. Bandpass filtering was then applied to the functional data, using a low threshold of 0.009 Hz and a high threshold of 0.08 Hz; temporal filtering reduces physiological noise given the short TR (Birn, 2012). Last, data were denoised using CSF and white matter masks and timepoints with framewise displacement greater than 0.5 mm were censored from the data.

Pre-processed resting state data and censored timepoints were visually inspected to remove potential outliers. One participant of the original 20 had 237 censored timepoints (40.9%) and was thus removed from further analyses. The remaining 19 participants had low motion (mean censored timepoints = 3.1, range = 0–13). Quantity of censored time points was not significantly different between participants with higher vs. lower misophonia scores (*t*(17) = 1.196, *p* = 0.248).

##### Functional magnetic resonance imaging task

All task data were also pre-processed using Freesurfer’s FS-Fast pre-processing pipeline. Each run was motion corrected to the first timepoint of the run, and timepoints with movement over 1 mm were removed from the analysis. Motion corrected volumes were registered to each participant’s native space. Data were then smoothed using a 4 mm full-width/half-maximum Gaussian kernel and convolved with a canonical hemodynamic response function.

Contrasts were calculated using FS-Fast, specifically for phoneme articulation (P) minus finger-tapping (T), or P > T. The reverse contrast, T > P, was calculated by taking the negative activation of the P > T contrast. Masks of significant data were resampled to 1 mm isotropic voxels and then registered to each participant’s anatomical scan and resting-state native space. All analyses presented here use contrasts defined in the first task run unless otherwise noted.

### Defining regions of interest

#### Non-sensorimotor regions of interest

**FIGURE 1:**
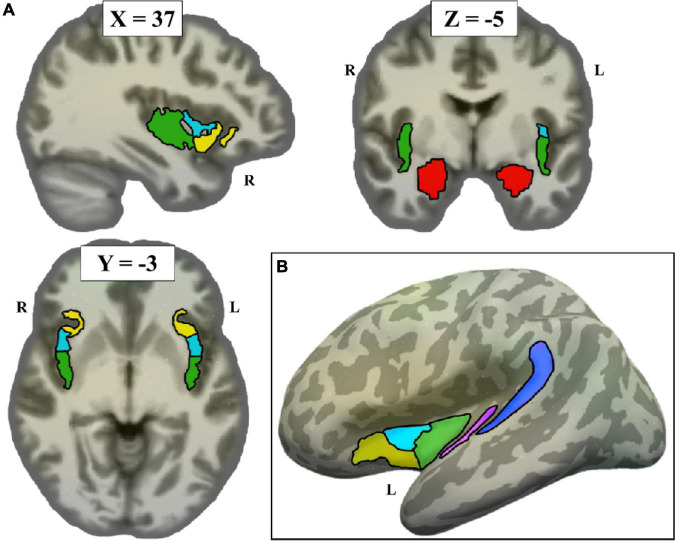
Non-sensorimotor regions of interest (ROIs), depicted on the Freesurfer CVS average-35 in MNI152 brain. **(A)** Volume view, at MNI coordinates (37, –3, –5). Red = amygdala, green = posterior insula, cyan = middle insula, yellow = anterior insula. **(B)** Surface view, showing all left hemisphere non-sensorimotor ROIs (minus amygdala) projected to the left inflated surface. Blue = planum temporale, pink = A1.

*A priori* ROIs from previous literature were defined anatomically in each participant’s native space (Figure 1), using both the Destrieux atlas (Destrieux et al., 2010) and the Glasser atlas (Glasser et al., 2016). The Destrieux atlas, defined in each participant’s native anatomical space through Freesurfer,^2^ was registered to each participant’s native resting-space. The Destrieux atlas was used to define primary auditory cortex (Heschl’s Gyrus, A1), secondary auditory cortex (Planum Temporale), and the amygdala. The Glasser atlas, originally obtained on the fsaverage surface, was transferred to each participant’s native resting-state space using Freesurfer. The Glasser atlas was used to define posterior, middle, and anterior subdivisions of the insula.

#### Defining motor masks

**FIGURE 2:**
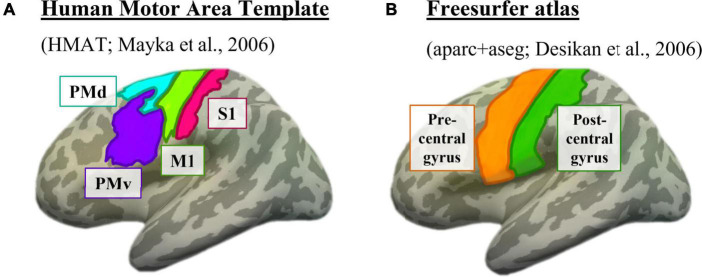
Motor masks, depicted on the left inflated surface of the Freesurfer CVS average-35 in MNI152 brain. **(A)** The four subdivisions of the Human Motor Area Template. Pink = primary somatosensory cortex (S1), lime green = primary motor cortex (M1), cyan = dorsal premotor cortex (PMd), purple = ventral premotor cortex (PMv). **(B)** The two sensorimotor subdivisions from the Freesurfer anatomical parcellation. Orange = motor strip (precentral gyrus), green = somatosensory strip (postcentral gyrus).

To attempt to theoretically replicate the motor finding from Kumar et al. (2021), we used their same method of overlaying a motor mask from the Human Motor Area Template (HMAT) (Mayka et al., 2006). The HMAT atlas was first registered from Talairach space to each participant’s native anatomical space, then registered to the participant’s resting-state native space. The HMAT atlas subdivided each participant’s sensorimotor area into four regions: primary somatosensory cortex (S1), primary motor cortex (M1), dorsal premotor area (PMd), and ventral premotor area (PMv) (Figure 2A).

Additionally, to expand the sensorimotor analysis and ensure that the use of a specific atlas did not influence the results, we used the Desikan Freesurfer parcellation in native anatomical space (Desikan et al., 2006) to identify the precentral and postcentral gyri (Figure 2B). These regions were chosen because of their canonical association with primary motor and primary somatosensory cortices, respectively. Since the precentral and postcentral gyri are defined using each individual’s anatomy as opposed to overlaying an atlas, it is possible this method will better capture individual nuances in cortical location.

#### Defining orofacial cortex

##### Method 1: Resting-state region of interest

First, we applied the method used by Kumar et al. (2021) to identify an orofacial resting-state region of interest (rsROI) in each individual. Specifically, we located the part of PMv that showed the strongest resting-state connectivity to the planum temporale. To do so, we averaged together the time courses of each voxel comprising the planum temporale, resulting in one vector representing the overall time course from the region. We then correlated that vector with the time course of each voxel within the PMv mask separately (for more detail, see “calculating functional connectivity” below), and sorted the connectivity values from largest to smallest. To maintain ROIs of similar sizes across analyses, we kept the top 10% of voxels from within PMv that had the highest connectivity to the planum temporale. This calculation was done in all nineteen participants individually, and the resulting voxels comprised that participant’s orofacial rsROI.

To explore the selectivity of connectivity differences to PMv specifically, we employed the same method to define an orofacial rsROI in each of S1, M1, and PMd as well. Additionally, for comparison, we used the Freesurfer anatomical atlas to define rsROIs in both the precentral and postcentral gyri.

##### Method 2: Functional region of interest

Next, we used the articulatory localizer fMRI task to subdivide sensorimotor cortex based on activation, from a scan independent of the resting-state data. We used the P > T contrast to identify regions representing physical orofacial movement (e.g., lips, jaw, tongue, throat, face) specifically. Because speech production overlaps considerably with effectors for orofacial movement generally (e.g., Takai et al., 2010; Kern et al., 2019) and speech sounds are a trigger reported in many studies specifically (e.g., Edelstein et al., 2013; Colucci, 2015; Claiborn et al., 2020; Cecilione et al., 2021), this localizer effectively accomplishes our goal of functionally defining an orofacial region relevant to misophonia. To maintain consistency with the rsROIs, we defined orofacial functional regions of interest (fROIs) within each mask (S1, M1, PMd, PMv, precentral gyrus, postcentral gyrus) as the top 10% of voxels within each region comprising the t-statistic’s positive tail (see Blank et al., 2014).

To explore whether the connectivity differences in misophonia were specific to orofacial cortex, we additionally defined finger cortex since finger-tapping has been described in previous literature as a common misophonic trigger (e.g., Cavanna and Seri, 2015). For instance, 58.7% of participants in a large-scale study of misophonia endorsed finger actions (i.e., snapping, tapping, or rubbing) as triggering (Claiborn et al., 2020), and “finger tapping” was ranked as the 15th most triggering item (out of 48 total) in a separate sample of 143 individuals with misophonia (Rinaldi et al., 2021).

Finger fROIs were defined in each participant using the negative tail of the P > T contrast to isolate cortical regions associated with finger movement. As with the orofacial fROIs, finger fROIs were defined as the top 10% of voxels active within each region. For a depiction of fROI locations, see Supplementary Figure 1.

**FIGURE 3:**
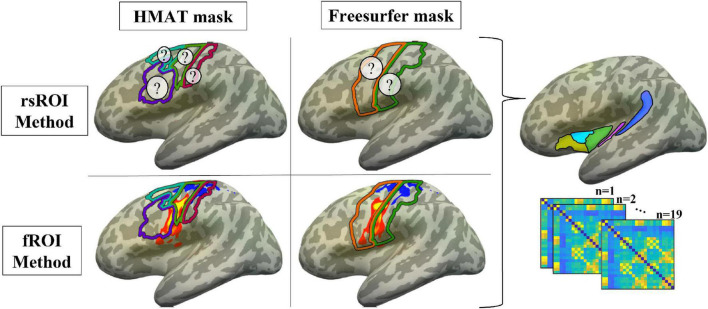
Methods summary. ROIs were defined using either the voxels most connected to planum temporale in resting state (rsROI Method, top row; ? represents unknown location of the most connected voxels) or the most activated voxels for the articulatory localizer fMRI task (fROI Method, bottom row; P-T activation in sensorimotor cortex for a representative participant, projected to the surface of the Freesurfer CVS average-35 in MNI152 brain for visualization; warm colors = P > T, cool = T > P). For each method, regions were defined within either the Human Motor Area Template mask (HMAT, left column; pink outline = S1, lime green = M1, cyan = PMd, purple = PMv) or Freesurfer parcellation (right column; orange outline = precentral gyrus, green = postcentral gyrus). Resting state data from all four sets of ROIs were correlated with data from the *a priori* non-sensorimotor ROIs (see Figure 1), creating functional connectivity matrices for each participant.

For an overview schematic of the ROI methods and sensorimotor templates being used in these analyses, see Figure 3.

### Analyses

#### Calculating percent signal change

Percent signal change (PSC) was calculated to assess ROI selectivity to either phoneme articulation or finger-tapping. PSC analyses were done in each participant’s anatomical brain.

For fROI selectivity, fROIs were defined using one run of the articulatory localizer fMRI task as described above. To avoid double-dipping within the same data (Kriegeskorte et al., 2009), PSCs were determined for each fROI using an independent task run; as such, only participants with two runs of the articulatory task (*n* = 11) were included in these analyses. To calculate PSC, the beta weights of phoneme articulation or finger tapping were divided by baseline and multiplied by 100. PSCs were calculated in each run separately (e.g., define fROI in run 1, calculate PSC in run 2; define fROI in run 2, calculate PSC in run 1) and averaged across both runs.

For rsROI selectivity, rsROIs were defined using resting-state connectivity as described above. PSCs were calculated in each rsROI using each run, then averaged across both runs.

#### Calculating functional connectivity

The mean time course of each non-sensorimotor ROI, rsROI, and fROI was computed from the pre-processed resting-state images. All ROIs were masked prior to calculations to only include voxels located in gray matter. Functional connectivity was calculated using Pearson’s correlations between the time courses of the non-sensorimotor ROIs and each orofacial/finger target region within each participant. To generate normally distributed values, each functional connectivity value was Fisher z-transformed.

Connectivity differences were analyzed using 3- and 4-way mixed ANOVAs, with group (two levels: higher vs. lower misophonia score) as a between-subject variable and non-sensorimotor ROI seed (six levels: A1, planum temporale, and amygdala; posterior, middle, and anterior insula) and orofacial/finger sensorimotor target (levels depending on method) as within-subject variables. Since significant hemispheric differences in connectivity patterns were not observed, ROIs were collapsed across hemispheres for the statistics and graphs reported here. Paired *t*-tests were conducted for within-group comparisons and independent *t*-tests for between-group comparisons. To correct for multiple comparisons, we used the Holm–Bonferroni method (Holm, 1979) to control the familywise Type I error rate (corrected *p*-values are denoted by *p*_HB_, uncorrected *p*-values are additionally provided to aid in interpretation).

## Results

### Analysis 1: Resting-state region of interest method

First, we sought to theoretically replicate the finding of Kumar et al. (2021) by defining orofacial cortex using resting state connectivity (i.e., rsROIs). Based on their results, we expected to see increased resting-state connectivity in individuals with higher misophonia scores between the PMv rsROI and both the planum temporale and insula.

#### Human Motor Area Template atlas

**FIGURE 4:**
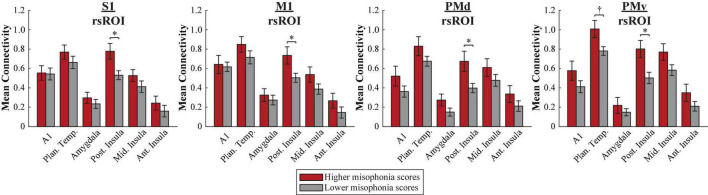
Functional connectivity between each HMAT rsROI and each non-sensorimotor ROI. Red bars = average connectivity across the seven participants with higher misophonia scores. Gray bars = average connectivity across the 12 participants with lower misophonia scores. Error bars are standard error of the mean. ^†^*p*_HB_ < 0.1, *p_HB_ < 0.05.

A 2 (group: higher vs. lower misophonia score) × 6 (non-sensorimotor ROI: A1, planum temporale, and amygdala; posterior, middle, and anterior insula) × 4 (rsROI: S1, M1, PMd, PMv) mixed ANOVA was conducted to assess sensorimotor connectivity differences associated with misophonia (Figure 4). There was a significant main effect of group (*F*(1,408) = 53.345, *p* = 1.481 × 10^–12^), such that individuals with higher misophonia scores had increased connectivity overall between these pre-selected regions than individuals with lower misophonia scores. Additionally, there was a significant anatomical ROI × rsROI interaction (*F*(15,408) = 2.145, *p* = 0.008). Pre-planned independent samples *t*-tests for each non-sensorimotor ROI–rsROI pairing revealed marginally significant group differences in connectivity between the PMv rsROI and the planum temporale (*t*(17) = 2.556, *p* = 0.020, *p*_HB_ = 0.082) and between the PMv rsROI and posterior insula (*t*(17) = 2.934, *p* = 0.009, *p*_HB_ = 0.037), as predicted. The posterior insula also showed significant group differences in connectivity with the S1 rsROI (*t*(17) = 2.876, *p* = 0.011, *p*_HB_ = 0.037), M1 rsROI (*t*(17) = 2.542, *p* = 0.021, *p*_HB_ = 0.028), and PMd rsROI (*t*(17) = 2.740, *p* = 0.014, *p*_HB_ = 0.032). No other connectivity pairings showed significant differences between groups (see Supplementary Table 2).

**FIGURE 5:**
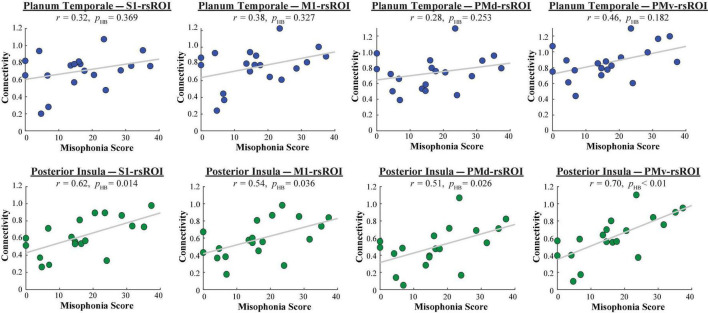
Functional connectivity between each HMAT rsROI and planum temporale (blue, top row) or posterior insula (green, bottom row), as a function of misophonia score.

To explore whether the planum temporale–rsROI or posterior insula–rsROI connectivity varied by misophonia severity, misophonia scores from all 19 participants were correlated with the connectivity values from each pairing (Figure 5). Whereas the four planum temporale pairings did not significantly correlate with misophonia scores after correction for multiple comparisons, misophonia level did significantly correlate with each of the four posterior insula pairings (S1: *r* = 0.62, *p* = 0.005, *p*_HB_ = 0.014; M1: *r* = 0.54, *p* = 0.018, *p*_HB_ = 0.036; PMd: *r* = 0.51, *p* = 0.026, *p*_HB_ = 0.026; PMv: *r* = 0.70, *p* = 9.147 × 10^–4^, *p*_HB_ = 3.659 × 10^–3^). To ensure this result was not better explained by demographic or psychopathological differences outside of misophonia, seven measures (OCD, depression, anxiety, stress, age, gender, race) were additionally used as nuisance regressors in a linear model, creating a “pure” metric of misophonia that excluded variance explained by these other variables. Connectivity was then correlated with this “pure” misophonia level as above. Misophonia still uniquely correlates with posterior insula–rsROI connectivity in all four pairings (S1: *r* = 0.52, *p* = 0.023, *p*_HB_ = 0.084; M1: *r* = 0.53, *p* = 0.021, *p*_HB_ = 0.084; PMd: *r* = 0.41, *p* = 0.085, *p*_HB_ = 0.085; PMv: *r* = 0.49, *p* = 0.035, *p*_HB_ = 0.070). As such, the original metric of misophonia will be used hereafter for simplicity.

Since non-parametric tests can additionally address any issues with smaller samples sizes, we constructed null distributions of possible t-statistics/correlations. We did so by randomly shuffling either group membership or misophonia scores, respectively, 5,000 times, and recalculating the t-statistics/correlation with functional connectivity that would have resulted each time. Each of the significant results mentioned here passed permutation testing (5,000 permutations, *p* < 0.05).

In sum, an rsROI defined within the PMv region of the HMAT atlas showed increased connectivity to planum temporale and insula in individuals with higher misophonia scores, matching what was found in Kumar et al. (2021). Additionally, rsROIs defined within S1, M1, and PMd also showed increased connectivity to the posterior insula, reflected in both significant differences in connectivity group means and significant correlations with misophonia scores.

#### Freesurfer atlas

**FIGURE 6:**
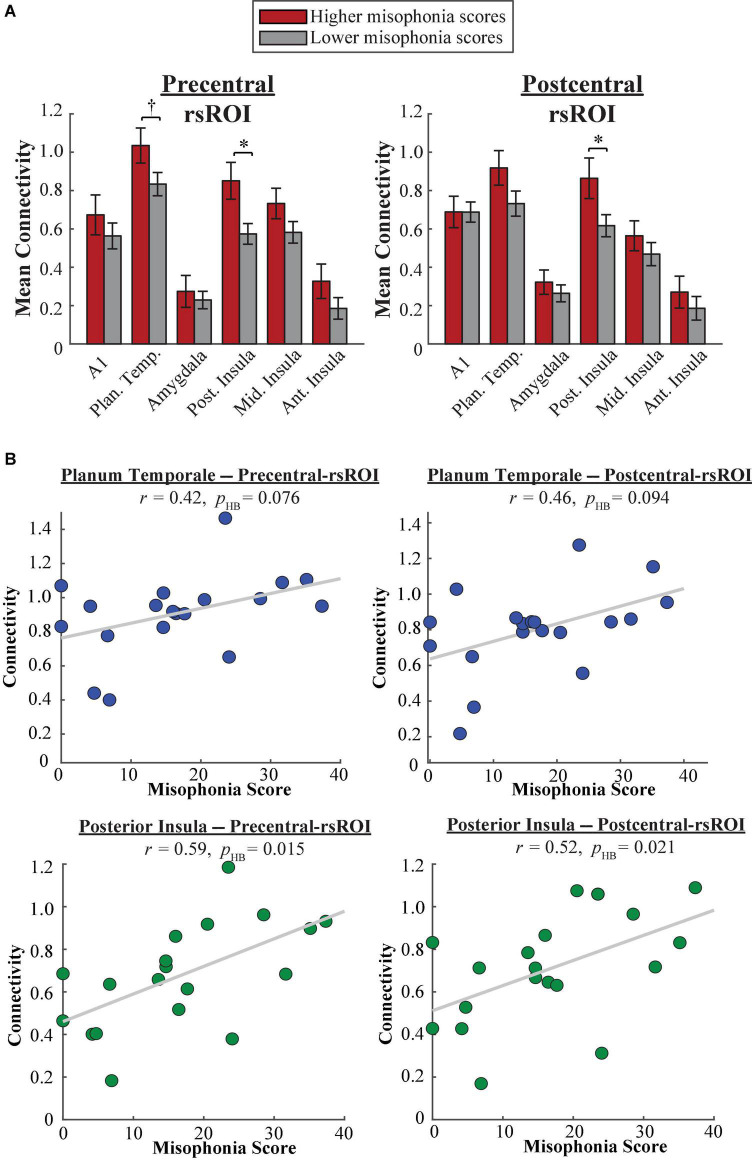
**(A)** Functional connectivity between each Freesurfer rsROI and each non-sensorimotor ROI. Red bars = average connectivity across the participants with higher misophonia scores. Gray bars = average connectivity across the participants with lower misophonia scores. Error bars are standard error of the mean. ^†^*p*_HB_ < 0.1, *p_HB_ < 0.05. **(B)** Functional connectivity between each Freesurfer rsROI and planum temporale (blue, top row) or posterior insula (green, bottom row), as a function of misophonia score.

A 2 (group: higher vs. lower misophonia score) × 6 (non-sensorimotor ROI: A1, planum temporale, and amygdala; posterior, middle, and anterior insula) × 2 (rsROI: precentral vs. postcentral gyrus) mixed ANOVA was conducted to assess sensorimotor connectivity differences associated with misophonia (Figure 6A). There was a significant main effect of group (*F*(1,204) = 21.107, *p* = 7.600 × 10^–6^), such that individuals with higher misophonia scores had increased connectivity overall between these pre-selected regions than individuals with lower misophonia scores. Although interactions were not significant, pre-planned independent samples *t*-tests for each non-sensorimotor ROI–rsROI pairing revealed a marginal uncorrected group difference in connectivity between the precentral rsROI and the planum temporale (*t*(17) = 1.907, *p* = 0.074, *p*_HB_ = 0.147). As with the HMAT atlas, posterior insula connectivity was significantly different between groups for both sensorimotor rsROIs (precentral: *t*(17) = 2.733, *p* = 0.014, *p*_HB_ = 0.028; postcentral: *t*(17) = 2.249, *p* = 0.038, *p*_HB_ = 0.038). Additionally, misophonia scores were positively correlated with connectivity from these areas, marginally so for planum temporale (precentral: *r* = 0.42, *p* = 0.076, *p*_HB_ = 0.076; postcentral: *r* = 0.46, *p* = 0.047, *p*_HB_ = 0.094) and significantly so for the posterior insula (precentral: *r* = 0.59, *p* = 0.008, *p*_HB_ = 0.015; postcentral: *r* = 0.52, *p* = 0.021, *p*_HB_ = 0.021) (Figure 6B). See Supplementary Table 3 for a complete list of results.

In sum, rsROIs defined within the precentral and postcentral gyri showed a similar pattern of connectivity to planum temporale as what would be expected from results of Kumar et al. (2021). Additionally, as with our HMAT analysis, both rsROIs showed increased connectivity to the posterior insula, reflected in both significant differences in connectivity group means and significant correlations with misophonia scores.

### Analysis 2: Functional region of interest method

We were able to show, using our sample of 19 participants from the general population, that individuals with higher misophonia scores do in fact show greater resting-state connectivity between the PMv rsROI and both the planum temporale and insula. However, a critical question remains: is the “orofacial” region defined using resting-state connectivity *really* an orofacial area? Or, in other words, how do the functionally defined orofacial and finger fROIs connect to the planum temporale and insula?

#### Human Motor Area Template atlas

**FIGURE 7:**
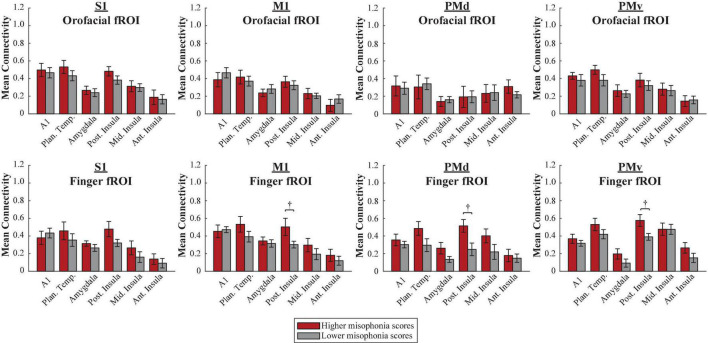
Functional connectivity between each HMAT fROI (top row = Orofacial, bottom row = Finger) and each non-sensorimotor ROI. Red bars = average connectivity across the participants with higher misophonia scores. Gray bars = average connectivity across the participants with lower misophonia scores. Error bars are standard error of the mean. ^†^*p*_HB_ < 0.1.

A 2 (group: higher vs. lower misophonia score) × 6 (non-sensorimotor ROI: A1, planum temporale, and amygdala; posterior, middle, and anterior insula) × 4 (HMAT region: S1, M1, PMd, PMv) × 2 (fROI: orofacial vs. finger) mixed ANOVA was conducted to assess sensorimotor connectivity differences associated with misophonia with either orofacial or finger cortex (Figure 7). There was a significant main effect of group (*F*(1,816) = 20.905, *p* = 5.575 × 10^–6^), such that individuals with higher misophonia had increased connectivity overall between these pre-selected regions than individuals with lower misophonia scores. Additionally, there was a significant group × fROI interaction (*F*(1,816) = 8.201, *p* = 0.004). Probing further, there was a significant main effect of fROI within the higher misophonia group (*F*(1,288) = 7.818, *p* = 0.006) but not within the lower misophonia group (*F*(1,528) = 0.929, *p* = 0.336). This result revealed that individuals with higher misophonia scores had greater connectivity with finger fROIs than with orofacial fROIs, but individuals with lower misophonia scores showed no difference between orofacial and finger connectivity.

Further, pre-planned independent samples *t*-tests for each non-sensorimotor ROI–fROI pairing revealed only uncorrected group differences in connectivity between the posterior insula and finger fROIs (M1-Finger: *t*(17) = 2.260, *p* = 0.037, *p*_HB_ = 0.224; PMd-Finger: *t*(17) = 2.439, *p* = 0.026, *p*_HB_ = 0.182; PMv-Finger: *t*(17) = 2.615, *p* = 0.018, *p*_HB_ = 0.145); no connections with orofacial fROIs nor with planum temporale were statistically significant, with or without corrections for multiple comparisons (see Supplementary Table 4).

In sum, individuals with higher misophonia scores showed more connectivity between the non-sensorimotor ROIs and finger fROIs than with orofacial fROIs, a result unique to higher misophonia scores only. Additionally, neither orofacial fROIs nor finger fROIs showed significant connectivity with planum temporale in misophonia, and only finger fROIs showed trending connectivity with posterior insula.

#### Freesurfer atlas

**FIGURE 8:**
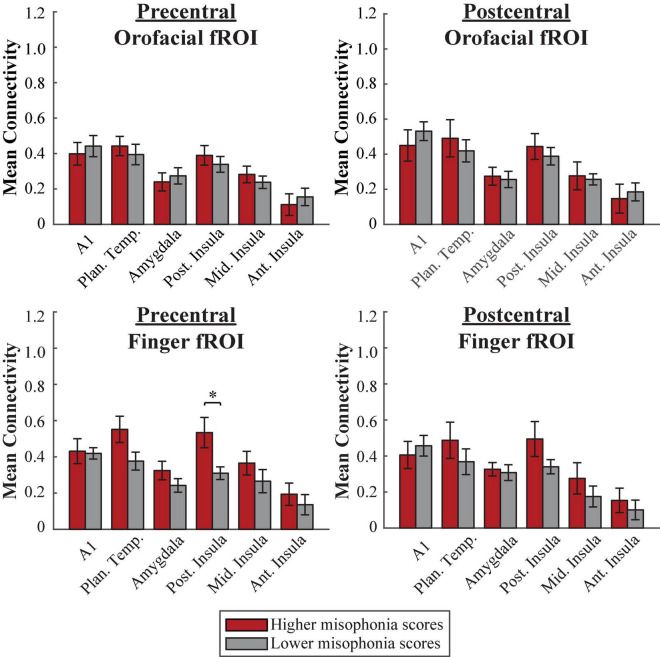
Functional connectivity between each Freesurfer fROI (top row = Orofacial, bottom row = Finger) and each non-sensorimotor ROI. Red bars = average connectivity across the participants with higher misophonia scores. Gray bars = average connectivity across the participants with lower misophonia scores. Error bars are standard error of the mean. *p_HB_ < 0.05.

A 2 (group: higher vs. lower misophonia score) × 6 (non-sensorimotor ROI: A1, planum temporale, and amygdala; posterior, middle, and anterior insula) × 2 (Freesurfer region: precentral vs. postcentral gyrus) × 2 (fROI: orofacial vs. finger) mixed ANOVA was conducted to assess sensorimotor connectivity differences associated with misophonia with either orofacial or finger cortex (Figure 8). There was a significant main effect of group (*F*(1,408) = 6.971, *p* = 0.009), such that individuals with higher misophonia scores had increased connectivity overall between these pre-selected regions than individuals with lower misophonia scores. Additionally, there was a significant group × fROI interaction (*F*(1,408) = 5.389, *p* = 0.021), although both groups showed only a marginal main effect of fROI (higher misophonia group: *F*(1,144) = 2.856, *p* = 0.093; lower misophonia group: *F*(1,264) = 2.357, *p* = 0.126).

Pre-planned independent samples *t*-tests for each non-sensorimotor ROI–fROI pairing revealed only a significant group difference for connectivity between the posterior insula and the precentral-finger fROI (*t*(17) = 2.882, *p* = 0.010, *p*_HB_ = 0.041); no connections with orofacial fROIs nor with planum temporale were statistically significant, with or without corrections for multiple comparisons (see Supplementary Table 5).

In sum, as with the HMAT atlas, neither orofacial fROIs nor finger fROIs defined within the Freesurfer atlas showed significant connectivity with planum temporale in misophonia. However, individuals with higher misophonia scores did show significantly more connectivity between the precentral-finger fROI and posterior insula than individuals with lower misophonia scores.

### Region of interest selectivity

As evidenced by the fROI method, the true orofacial motor regions do not show the same pattern of connectivity results that the rsROI method did. Are these previously used rsROIs selective for orofacial movement? To investigate the differences between these ROI methods further, we first compared the degree of overlap between each participant’s rsROI and corresponding fROIs. For each HMAT region, the proportion of fROI overlap was calculated for each participant by dividing the number of voxels in common to both the rsROI and the fROI by the number of voxels of the entire fROI. Overall, the proportion of overlap was low across all regions (*M* = 0.106, *SD* = 0.021, range = 0.000–0.455) and did not vary systematically with misophonia level, nor was it significantly different between fROIs. Sparse overlap demonstrates that the rsROIs are not capturing the most selective voxels for either orofacial or finger regions.

Do the rsROIs show any preference for orofacial (or finger) movement at all? For each HMAT rsROI, PSC was calculated using the articulatory localizer fMRI task to determine whether the voxels comprising the rsROI showed an increase in activation to either phoneme articulation or finger-tapping. For a comparison, PSC was also calculated within each fROI, using independent runs from what was used to define the fROI.

**FIGURE 9:**
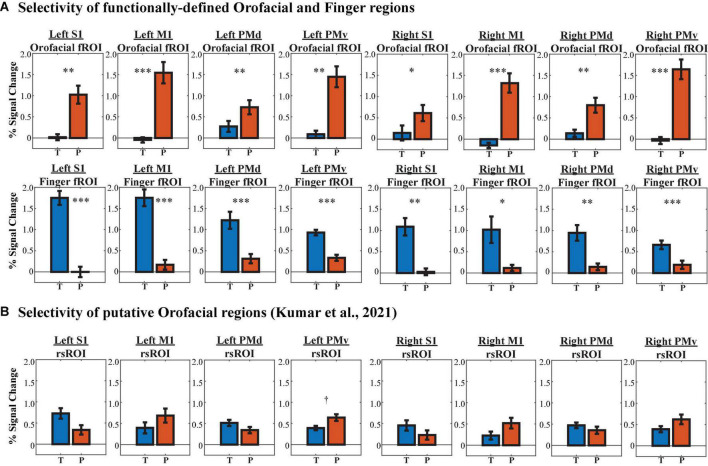
Region of interest (ROI) selectivity to phoneme production (P, red bars) vs. finger-tapping (T, blue bars) in each HMAT region, determined via percent signal change from baseline activation. **(A)** Selectivity of orofacial fROIs (1st row) and finger fROIs (2nd row). **(B)** Selectivity of rsROIs described by Kumar et al. (2021) to represent orofacial function. ^†^*p*_HB_ < 0.1, *p_HB_ < 0.05, ^**^p_HB_ < 0.01, ^***^p_HB_ < 0.001.

First, a 2 (localizer activation: phoneme production vs. finger-tapping) × 4 (HMAT region: S1, M1, PMd, PMv) × 2 (hemisphere: left vs. right) within-group ANOVA was conducted to assess differences in functional selectivity within each HMAT fROI (Figure 9A). There was a significant main effect of hemisphere (*F*(1,351) = 10.095, *p* = 0.002), such that left hemisphere fROIs showed greater PSC regardless of task or HMAT region. Additionally, there was a significant activation × region interaction (*F*(7,351) = 55.918, *p* = 6.927 × 10^–52^). To explore further, paired *t*-tests were calculated between phoneme vs. finger activation within each HMAT fROI. When corrected for multiple comparisons, all sixteen fROIs showed significant selectivity for their respective localizer task. Thus, the fROIs are reliably capturing the function they were intended to represent.

Are the rsROIs, which were previously attributed to orofacial function by Kumar et al. (2021), actually selective for orofacial actions (i.e., phoneme production)? As above, a 2 (localizer activation: phoneme production vs. finger-tapping) × 4 (HMAT region: S1, M1, PMd, PMv) × 2 (hemisphere: left vs. right) within-group ANOVA was conducted to assess differences in functional selectivity within each HMAT rsROI (Figure 9B). The main effect of hemisphere was marginal, with higher activation in the left hemisphere rsROIs overall (*F*(1,175) = 3.333, *p* = 0.070), and the activation × region interaction was significant (*F*(3,17) = 7.855, *p* = 6.364 × 10^–5^). However, paired *t*-tests between phoneme vs. finger activation within each HMAT rsROI revealed little task selectivity; only one of the eight rsROIs showed any preference for phoneme production, and it was marginal after correcting for multiple comparisons (*t*(10) = 3.230, *p* = 0.009, *p*_HB_ = 0.072).

## Discussion

What is the underlying neural basis of misophonia? In the present analyses, we show that an ROI within PMv created using resting-state connectivity to planum temporale (as well as the entire PMv region as an ROI, see Supplementary Figure 3) conceptually replicates prior findings (Kumar et al., 2021). This rsROI showed increased connectivity to planum temporale and insula in individuals with higher misophonia scores, corroborating previous neuroimaging findings that auditory cortex and insula are key regions whose connectivity differentiates misophonia from controls (Kumar et al., 2017, 2021; Schröder et al., 2019). Of note, although previous literature has described group differences in the anterior insula, the corresponding coordinates of maxima activation/connectivity fall closer to the posterior insula ROI used in this study; we do not see this as an incompatible finding, but rather an artifact of using different anatomical atlases across the misophonia literature (e.g., Kumar et al., 2017, 2021 used the Neuromorphometrics SPM toolbox; Schröder et al., 2019 used the Anatomical Automatic Labeling toolbox). Additionally, the present results lend credence to the potential involvement of the posterior insula in misophonia, supported by previous findings linking the posterior insula to sensorimotor and auditory processes (Uddin et al., 2017).

Moreover, although we observed a main effect of group, it is not the case that individuals with higher misophonia had higher connectivity with all of our pre-selected non-sensorimotor ROIs: the high vs. low misophonia groups showed no difference in connectivity of A1, supporting previous findings that misophonia is not merely a disorder of lower-level sound properties (Edelstein et al., 2020; Kumar et al., 2021). Our finding was specific to the planum temporale and insula, supporting theories that the abnormalities associated with misophonia concern more higher-level perceptions/context of the sound (Swedo et al., 2021) and personally assigned salience (Schröder et al., 2019). However, using rsROIs created in additional sensorimotor areas and fROIs created from independent task fMRI, we provide evidence that this finding is neither exclusive to (a) motor function, nor (b) orofacial content.

Constraining “orofacial” cortex by using only the voxels within PMv misses out on important non-motor function that may be equally informative to deciphering the mechanism underlying misophonia. For instance, a study investigating the existence of a mirror system in PMv during the observation of mouth actions (e.g., biting an apple, chewing) vs. hand actions (e.g., grasping a cup) notes that, in addition to premotor cortex activation, observation of both mouth and hand actions elicited activation in the inferior parietal lobule (Buccino et al., 2013), a region thought to integrate higher-order sensory and motor information (Fogassi and Luppino, 2005). Moreover, other studies have shown that activation during orofacial/finger tasks can be found both dorsally (Meister et al., 2009) and ventrally (orofacial: Grabski et al., 2012; finger: Ruspantini et al., 2011) in the premotor cortex. By similarly defining an rsROI within each of the four HMAT parcels, we were able to investigate the specificity of the Kumar et al. (2021) finding to ascertain whether differences were unique to PMv. Contrary to the Kumar et al. (2021) conclusion, our analyses showed significantly higher connectivity in misophonia between the insula and rsROIs defined within all four HMAT regions (S1, M1, PMd, PMv). This is noteworthy for a few reasons: First, differences outside of just PMv limit the viability of mirror neurons as being the causal instigator of misophonia, given that mirror neurons are thought to be mainly located in PMv/area F5 (Fabbri-Destro and Rizzolatti, 2008). Second, our current findings of differential insular connectivity to rsROIs created within S1 and the postcentral gyrus, combined with the parietal lobe activation found in previous work, suggest potential sensory (not just motor) mechanisms that may underlie misophonia.

Further, for the first time to our knowledge, the present experiment provides a possible neural substrate for the non-orofacial triggers in misophonia. Using fROIs constructed from participants tapping their fingers in the scanner, we find that these finger regions–both in motor and somatosensory areas–show significant differences in connectivity to the insula in individuals with higher misophonia scores. If misophonia was a condition of aversion to solely (or primarily) oral/nasal triggering stimuli, there would not have been any reason to see systematic differences in connectivity between insula and finger regions. However, neural differences to finger regions seem plausible, given the plethora of non-oral/nasal misophonia triggers that are made using the fingers, either alone (e.g., finger-tapping; Cavanna and Seri, 2015; Claiborn et al., 2020) or with an object (e.g., typing on a keyboard, clicking a pen, clicking a mouse, etc.; Edelstein et al., 2013; Hansen et al., 2021). Moreover, prior work using magnetoencephalography (MEG) has shown similar neural representations in human primary motor cortex when participants tap a drum with their finger as when they observe or hear the drum being tapped (Caetano et al., 2007), demonstrating an involvement of finger motor cortex in finger-related sounds. To briefly address the existence of non-orofacial triggers, Kumar et al. (2021) posited that other triggers are acquired through associative learning after the orofacial trigger is acquired, without allowing for the possibility that non-orofacial triggers (like finger-tapping) might also be neurally represented. As a whole, our finding casts doubt on this explanation and supports direct neural representation for non-orofacial triggers.

Additionally, given the low overlap between the rsROIs and the fROIs used in this experiment (see Supplementary Figure 2 for a depiction of PMv ROIs) and the low selectivity of the rsROIs in general, there is doubt as to what the function(s) of the voxels comprising the rsROIs actually are. It would appear that the voxels most strongly connected to the planum temporale in resting state are neither entirely orofacial nor entirely finger voxels; if they were, we would expect to find some task-based selectivity of these voxels to either phoneme production or finger-tapping. This finding opens the door to discovery of what those rsROIs are actually responsive or selective to, perhaps illuminating a more nuanced mechanism to misophonia than just “mirroring” the production of triggers.

It is worth noting that the participants we studied were members of the general population, not specifically misophonia-sufferers. They were not recruited (or excluded) for having particular misophonic triggers. The participants varied in their identification with misophonic experiences, demonstrating the commonality of mild misophonia in the general population (Wu et al., 2014; Zhou et al., 2017). However, the sample of individuals with higher misophonia scores was comparatively small and experienced less severe misophonia than the misophonia sample in Kumar et al. (2021) similarly, the individuals with lower misophonia scores were not a true control group. Despite these weaknesses, our analyses still showed significant between-group connectivity differences even with groups more similar in misophonia severity. A replication of the rsROI method within this sample lends credence to the power of the data to reveal group differences, if they existed. Further, correlating misophonia severity with connection strength between the insula and sensorimotor regions revealed that these connections are systematically stronger with worse misophonia severity. Nevertheless, future studies should seek to incorporate individuals with more extreme misophonic discomfort in larger sample sizes to ascertain stronger group differences.

Regardless, the present results have important implications for the study of misophonia moving forward. As we have previously argued, misophonia ought to be conceptualized as more than just an aversion to oral/nasal sounds (Hansen et al., 2021). Neural evidence provided here of abnormal connectivity to functionally defined finger regions underlines this point. Further, these results urge an expanded view of the underlying mechanisms of misophonia. (Kumar et al., 2021) discovered that connectivity within motor cortex differed in misophonia, and the data presented here expands this mechanism by showing differences in sensory cortex, too; thus, both motor and sensory routes should be studied further as possible misophonia explanations. Taken together, these results take us one step closer to understanding the multitude of presentations of which misophonia likely exists, which is crucial for inclusive diagnosis and treatment.

## Data availability statement

The raw data supporting the conclusions of this article will be made available by the authors, without undue reservation.

## Ethics statement

The studies involving human participants were reviewed and approved by The Ohio State University Institutional Review Board. The patients/participants provided their written informed consent to participate in this study.

## Author contributions

HH conceptualized the project, analyzed the data, and prepared the manuscript. PS recruited and collected the data for the experiment, pre-processed the data, and provided feedback on the manuscript. ZS wrote the experiment and pre-processing scripts. AL and ZS supervised the project, assisted with analyses, and provided edits and feedback on the manuscript. All authors contributed to the article and approved the submitted version.

## Footnotes

1
https://surfer.nmr.mgh.harvard.edu/fswiki/FsFastAnlysisBySteps

2
https://surfer.nmr.mgh.harvard.edu/fswiki/recon-all
