# Supplementary material for: Neural evidence for non-orofacial triggers in mild misophonia
Source: Front Neurosci. 2022 Aug 9;16:880759. doi: 10.3389/fnins.2022.880759 (PMC9397125; doi:10.3389/fnins.2022.880759)
Supplement: Supplementary file 1 [file Data_Sheet_1.pdf]

Supplementary Material

1 Misophonia Assessments

Supplementary Table 1. Demographics and average questionnaire scores for higher vs. lower misophonia. MAS-1, MAQ-2, and A-MISO-S columns depict mean values. Total rows show Mean (SD), unless otherwise noted.

Group	Gender	Age	MAS-1	MAQ-2	A-MISO-S	Composite	A-MISO-S Total Score	Suggested A-MISO-S Classification
**Higher Misophonia**	F	28.3	6.5	0.3	1.5	37.3	9.0	mild
	M	21.1	3.5	1.3	1.2	35.1	7.0	mild
	M	27.6	4.0	0.5	1.5	31.7	9.0	mild
	F	27.1	3.0	0.7	1.3	28.5	8.0	mild
	F	21.3	1.5	0.7	1.3	24.0	8.0	mild
	F	20.3	3.5	0.2	1.2	23.5	7.0	mild
	M	23.9	3.5	0.0	1.0	20.5	6.0	mild
**Total**	**4F (3M)**	**24.2 (3.4)**	**3.6 (1.5)**	**0.5 (0.4)**	**1.3 (0.2)**	**28.7 (6.3)**	**7.7 (1.1)**	**mild misophonia**
**Lower Misophonia**	F	33.2	3.0	0.2	0.7	17.7	4.0	subclinical
	F	27.4	2.0	0.4	0.7	16.5	4.0	subclinical
	M	36.0	2.5	0.2	0.7	16.0	4.0	subclinical
	F	18.7	3.5	0.1	0.2	14.6	1.0	subclinical
	F	20.0	2.5	0.2	0.5	14.6	3.0	subclinical
	F	19.3	2.5	0.1	0.5	13.6	3.0	subclinical
	M	20.6	1.5	0.0	0.2	6.9	1.0	subclinical
	F	22.1	1.0	0.0	0.3	6.6	2.0	subclinical
	F	26.3	1.0	0.0	0.2	4.7	1.0	subclinical
	M	26.8	0.0	0.0	0.5	4.2	3.0	subclinical
	F	20.6	0.0	0.0	0.0	0.0	0.0	subclinical
	F	45.7	0.0	0.0	0.0	0.0	0.0	subclinical
**Total**	**9F (3M)**	**26.4 (8.2)**	**1.6 (1.2)**	**0.1 (0.1)**	**0.4 (0.3)**	**9.6 (6.6)**	**2.2 (1.5)**	**subclinical misophonia**

MAS-1: Misophonia Activation Scale (max score per question: 10; Fitzmaurice, 2014)
MAQ-2: Misophonia Assessment Questionnaire (max score per question: 3; Johnson & Dozier, 2013)
A-MISO-S: Amsterdam Misophonia Scale (max score per question: 4; max score total: 24; Schröder et al., 2013).
Composite formula: standardizes and equally weights all three assessments

$$\text{for each individual } i\text{: composite} = \frac{100}{3} \cdot \left(\frac{MAS1_i}{10} + \frac{MAQ2_i}{3} + \frac{AMISOS_i}{4} \right)$$

2 Average location of regions of interest

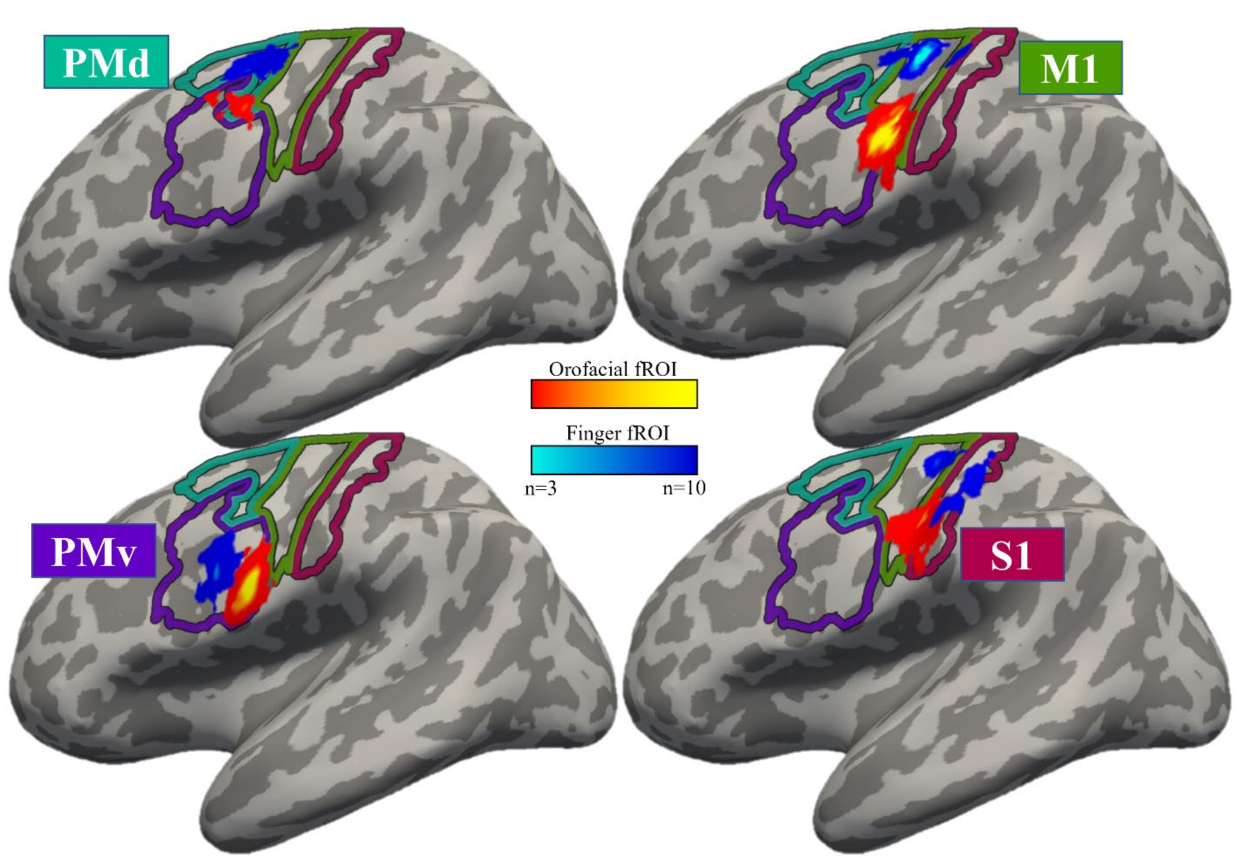

Supplementary Figure 1. Location of functionally-defined regions of interest (fROIs), averaged across all participants and projected to the surface of the Freesurfer CVS average-35 in MNI152 brain for visualization. Warm colors = Orofacial fROIs (phoneme articulation>fingertapping), cool colors = Finger fROIs (fingertapping>phoneme articulation). Given individual differences in fROI location, data depict locations where at least 3 participants had overlap. fROIs are shown separately for each HMAT region (pink/S1 = primary somatosensory cortex, lime green/M1 = primary motor cortex, cyan/PMd = dorsal premotor cortex, purple/PMv = ventral premotor cortex.

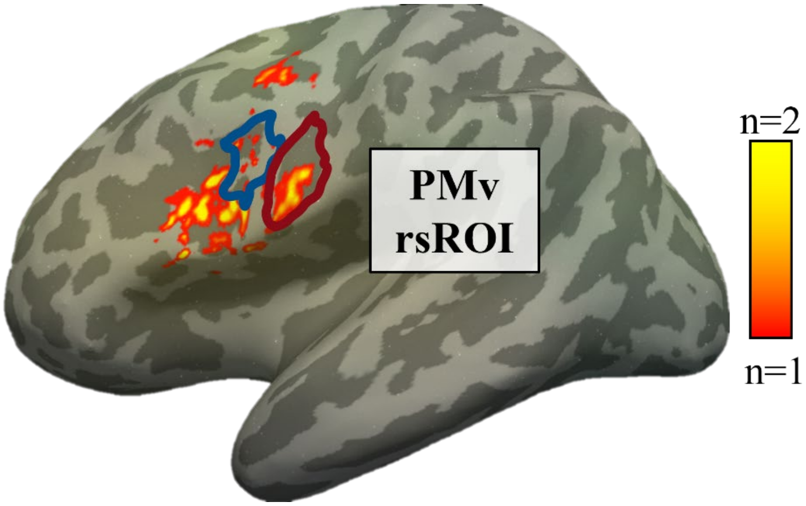

Supplementary Figure 2. Location of the resting-state region of interest (rsROI) defined within PMv, recreating what Kumar et al. (2021) called “orofacial cortex”. rsROIs were calculated in each individual, then averaged across all 19 participants and projected to the surface of the Freesurfer CVS average-35 in MNI152 brain for visualization. Note that given individual variability, no location in PMv had more than 2 participants’ PMv-rsROIs overlap. For comparison, red and blue outlines depict the average location of the Orofacial fROI and Finger fROI within PMv, respectively.

3 Additional Results

For each analysis in the main manuscript, we subdivide a region of interest within each motor mask (e.g., HMAT parcel). Analysis 1 subdivides by the voxels most connected to planum temporale in resting state. Analysis 2 subdivides by the voxels most active during a functional task. Since Kumar et al. (2021) additionally presents connectivity results to the entire PMv region, we sought to verify that we find similar connectivity from the entire PMv region as we do from the ROI analyses. Results showed significant group differences in connectivity between the entire PMv and the planum temporale ($t(17) = 2.779$, $p = 0.013$, $p_{HB} = 0.052$), as was found in Kumar et al. (2021).

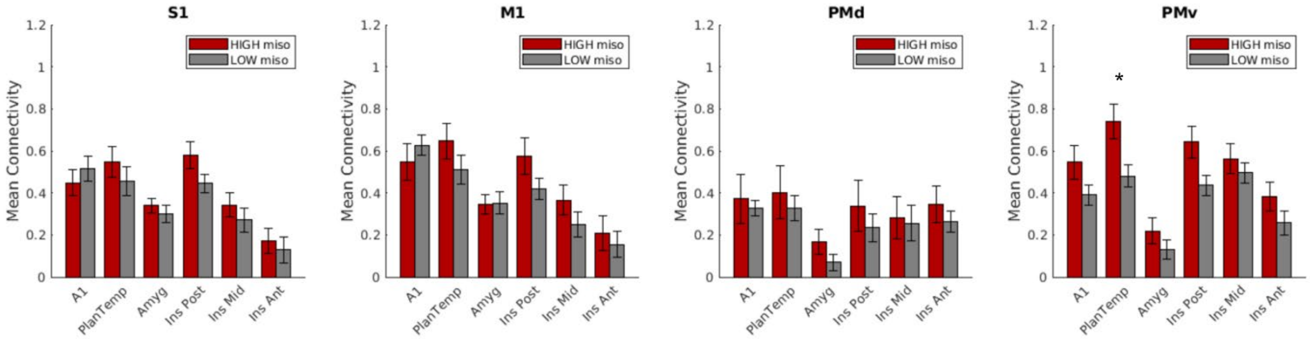

Supplementary Figure 3. Functional connectivity between the entire HMAT regions and each non-sensorimotor ROI. Red bars = average connectivity across the participants with higher misophonia scores. Gray bars = average connectivity across the participants with lower misophonia scores. Error bars are standard error of the mean.

Supplementary Table 2. Analysis 1: rsROI method, HMAT atlas

		higher vs. lower misophonia (t-test)			misophonia score vs. connectivity (correlation)		
**Non-sensorimotor ROI**	**HMAT rsROI**	***t***	***p***	p_{HB}	***r***	***p***	p_{HB}
A1	S1	0.105	0.918	1.592	-0.058	0.814	1.627
	M1	0.263	0.796	1.592	-0.018	0.941	1.627
	PMd	1.469	0.160	0.608	0.193	0.428	1.285
	PMv	1.500	0.152	0.608	0.271	0.263	1.050
Planum Temporale	S1	1.056	0.306	0.461	0.318	0.184	0.369
	M1	1.244	0.230	0.461	0.379	0.109	0.327
	PMd	1.555	0.138	0.415	0.276	0.253	0.369
	PMv	2.556	0.021	0.082	0.464	0.045	0.182
Amygdala	S1	0.827	0.420	1.135	0.141	0.564	1.354
	M1	0.621	0.543	0.840	0.059	0.812	1.127
	PMd	1.742	0.100	0.398	0.334	0.163	0.652
	PMv	0.904	0.378	1.135	0.184	0.451	1.354
Posterior Insula	S1	2.876	0.011	0.037	0.618	0.005	0.015
	M1	2.542	0.021	0.028	0.535	0.018	0.037
	PMd	2.740	0.014	0.032	0.510	0.026	0.037
	PMv	2.934	0.009	0.037	0.697	<0.001	0.004
Middle Insula	S1	1.263	0.224	0.447	0.301	0.211	0.440
	M1	1.639	0.120	0.359	0.346	0.147	0.440
	PMd	1.251	0.228	0.447	0.239	0.325	0.422
	PMv	1.899	0.075	0.299	0.464	0.045	0.181
Anterior Insula	S1	0.861	0.401	0.438	0.097	0.693	2.079
	M1	1.277	0.219	0.647	0.157	0.520	2.079
	PMd	1.286	0.216	0.647	0.045	0.855	1.469
	PMv	1.484	0.156	0.624	0.083	0.734	2.078

first columns: ROI pairing for which functional connectivity was calculated
middle columns: *t*-test results and corresponding *p*-values comparing mean connectivity between individuals with higher vs. lower misophonia scores for each pairing
last columns: Pearson's *r* correlations and corresponding *p*-values between connectivity and misophonia scores across all participants

Note: p-values are Holm-Bonferroni corrected (p_{HB}) within each non-sensorimotor seed.

Supplementary Table 3. Analysis 1: rsROI method, Freesurfer atlas

		**higher vs. lower misophonia (t-test)**			**misophonia score vs. connectivity (correlation)**		
**Non-sensorimotor ROI**	**Freesurfer rsROI**	***t***	***p***	p_{HB}	***r***	***p***	p_{HB}
A1	precentral	0.926	0.368	0.735	0.135	0.583	0.870
	postcentral	0.009	0.993	0.993	-0.190	0.435	0.870
Planum	precentral	1.907	0.074	0.147	0.416	0.076	0.094
Temporale	postcentral	1.694	0.109	0.147	0.461	0.047	0.094
Amygdala	precentral	0.521	0.609	0.902	0.089	0.716	1.433
	postcentral	0.772	0.451	0.902	0.065	0.793	1.433
Posterior	precentral	2.733	0.014	0.028	0.592	0.008	0.015
Insula	postcentral	2.249	0.038	0.038	0.524	0.021	0.021
Middle	precentral	1.580	0.133	0.265	0.277	0.251	0.501
Insula	postcentral	0.968	0.346	0.346	0.240	0.323	0.501
Anterior	precentral	1.410	0.177	0.353	0.126	0.607	1.215
Insula	postcentral	0.821	0.423	0.423	0.103	0.676	1.215

first columns: ROI pairing for which functional connectivity was calculated
middle columns: *t*-test results and corresponding *p*-values comparing mean connectivity between individuals with higher vs. lower misophonia scores for each pairing
last columns: Pearson's *r* correlations and corresponding *p*-values between connectivity and misophonia scores across all participants

Note: p-values are Holm-Bonferroni corrected (p_{HB}) within each non-sensorimotor seed.

Supplementary Table 4. Analysis 2: fROI method, HMAT atlas

			higher vs. lower misophonia (t-test)			misophonia score vs. connectivity (correlation)		
**Non-sensorimotor ROI**	**fROI**	**HMAT region**	***t***	***p***	***p*HB**	***r***	***p***	***p*HB**
A1	*Orofacial*	S1	0.325	0.750	2.353	0.046	0.851	2.577
		M1	-0.805	0.432	3.164	-0.165	0.499	3.064
		PMd	0.206	0.839	1.575	-0.189	0.438	3.064
		PMv	0.552	0.588	2.816	-0.002	0.995	1.787
	Finger	S1	-0.590	0.563	2.816	-0.235	0.333	2.660
		M1	-0.274	0.787	2.249	-0.140	0.567	2.991
		PMd	0.758	0.459	3.023	0.033	0.894	2.553
		PMv	0.872	0.396	3.164	0.113	0.644	2.837
Planum Temporale	*Orofacial*	S1	1.063	0.303	1.210	0.266	0.272	0.953
		M1	0.488	0.632	1.263	0.284	0.238	1.033
		PMd	-0.270	0.790	1.263	-0.212	0.383	0.718
		PMv	1.269	0.221	1.296	0.352	0.140	0.979
	Finger	S1	0.863	0.400	1.210	0.223	0.359	0.815
		M1	1.353	0.194	1.357	0.304	0.207	1.152
		PMd	1.673	0.113	0.902	0.313	0.192	1.152
		PMv	1.285	0.216	1.357	0.396	0.093	0.743
Amygdala	*Orofacial*	S1	0.392	0.700	1.984	0.050	0.840	1.572
		M1	-0.616	0.546	2.731	-0.169	0.490	2.448
		PMd	-0.329	0.747	1.400	-0.264	0.274	1.644
		PMv	0.503	0.621	2.731	-0.075	0.760	2.317
	Finger	S1	0.866	0.399	2.392	0.136	0.579	2.448
		M1	0.446	0.661	2.485	0.067	0.786	2.281
		PMd	1.928	0.071	0.566	0.376	0.113	0.904
		PMv	1.395	0.181	1.268	0.327	0.172	1.201
Posterior Insula	*Orofacial*	S1	1.342	0.197	0.789	0.292	0.226	0.904
		M1	0.493	0.628	1.523	0.272	0.259	0.904
		PMd	-0.007	0.994	1.257	-0.170	0.487	0.525
		PMv	0.677	0.508	1.523	0.271	0.262	0.778
	Finger	S1	1.869	0.079	0.395	0.366	0.123	0.819
		M1	2.260	0.037	0.224	0.372	0.117	0.819
		PMd	2.439	0.026	0.182	0.346	0.147	0.740
		PMv	2.615	0.018	0.145	0.531	0.019	0.154
Middle Insula	*Orofacial*	S1	0.203	0.842	3.366	-0.006	0.980	2.461
		M1	0.434	0.670	3.348	0.132	0.591	2.715

		PMd	-0.063	0.951	2.571	-0.242	0.319	2.550
		PMv	0.183	0.857	3.366	0.056	0.820	2.461
	Finger	S1	1.052	0.307	2.151	0.199	0.414	2.901
		M1	1.019	0.323	2.151	0.149	0.543	2.835
		PMd	1.434	0.170	1.358	0.176	0.473	2.901
		PMv	0.012	0.991	1.901	0.003	0.991	1.960
Anterior Insula	*Orofacial*	S1	0.245	0.809	2.153	0.042	0.866	3.230
		M1	-0.879	0.392	2.352	-0.099	0.686	3.910
		PMd	1.261	0.224	1.570	0.164	0.501	4.011
		PMv	-0.181	0.859	1.618	0.028	0.910	1.755
	Finger	S1	0.556	0.586	2.409	0.111	0.651	4.553
		M1	0.719	0.482	2.409	0.060	0.808	3.432
		PMd	0.368	0.718	2.342	-0.111	0.652	4.553
		PMv	1.372	0.188	1.504	0.038	0.878	2.598

first columns: ROI pairing for which functional connectivity was calculated
middle columns: *t*-test results and corresponding *p*-values comparing mean connectivity between individuals with higher vs. lower misophonia scores for each pairing
last columns: Pearson's *r* correlations and corresponding *p*-values between connectivity and misophonia scores across all participants

Note: *p*-values are Holm-Bonferroni corrected (p_{HB}) within each non-sensorimotor seed.

Supplementary Table 5. Analysis 2: fROI method, Freesurfer atlas

Non-sensorimotor ROI	fROI	Freesurfer region	higher vs. lower misophonia (t-test)			misophonia score vs. connectivity (correlation)		
			t	***p***	p_{HB}	***r***	***p***	p_{HB}
A1	*Orofacial*	precentral	-0.470	0.645	1.786	-0.156	0.523	1.686
		postcentral	-0.837	0.414	1.656	-0.115	0.640	1.570
	Finger	precentral	0.186	0.855	1.289	-0.026	0.915	1.281
		postcentral	-0.541	0.595	1.786	0.254	0.293	0.371
Planum Temporale	*Orofacial*	precentral	0.552	0.588	1.086	0.254	0.293	0.371
		postcentral	0.621	0.543	1.086	0.320	0.181	0.544
	Finger	precentral	2.056	0.056	0.222	0.430	0.066	0.264
		postcentral	0.986	0.338	1.015	0.318	0.185	0.544
Amygdala	*Orofacial*	precentral	-0.476	0.640	1.921	-0.185	0.449	1.347
		postcentral	0.255	0.802	1.555	<0.001	0.997	1.821
	Finger	precentral	1.312	0.207	0.829	0.273	0.258	1.031
		postcentral	0.287	0.778	1.921	-0.028	0.910	1.821
Posterior Insula	*Orofacial*	precentral	0.705	0.490	0.981	0.338	0.157	0.241
		postcentral	0.645	0.528	0.981	0.369	0.121	0.330
	Finger	precentral	2.882	0.010	0.041	0.492	0.032	0.129
		postcentral	1.723	0.103	0.309	0.379	0.110	0.330
Middle Insula	*Orofacial*	precentral	0.759	0.458	0.995	0.181	0.459	1.376
		postcentral	0.274	0.787	0.917	0.135	0.582	0.933
	Finger	precentral	1.017	0.324	1.294	0.178	0.467	1.376
		postcentral	1.000	0.332	1.294	0.239	0.325	1.298
Anterior Insula	*Orofacial*	precentral	-0.546	0.592	1.670	-0.058	0.815	2.444
		postcentral	-0.422	0.679	1.184	-0.002	0.995	1.867
	Finger	precentral	0.665	0.515	2.059	-0.021	0.933	2.444
		postcentral	0.600	0.557	2.059	0.129	0.598	2.391

first columns: ROI pairing for which functional connectivity was calculated
middle columns: *t*-test results and corresponding *p*-values comparing mean connectivity between individuals with higher vs. lower misophonia scores for each pairing
last columns: Pearson's *r* correlations and corresponding *p*-values between connectivity and misophonia scores across all participants

Note: p-values are Holm-Bonferroni corrected (p_{HB}) within each non-sensorimotor seed.
